# The Spectrum of Global Electron Content: A New Potential Indicator of Space Weather Activity

**DOI:** 10.3390/s24020393

**Published:** 2024-01-09

**Authors:** Josep Maria Aroca-Farrerons, Manuel Hernández-Pajares, Haixia Lyu, David Roma-Dollase, Raul Orus-Perez, Alberto García-Rigo, Victoria Graffigna, Germán Olivares-Pulido, Enric Monte-Moreno, Heng Yang, Qi Liu

**Affiliations:** 1 UPC-IonSAT, 08034 Barcelona, Spain; josep.m.aroca@upc.edu (J.M.A.-F.); hxlyu@whu.edu.cn (H.L.); alberto.garcia.rigo@upc.edu (A.G.-R.); victoria.graffigna@upc.edu (V.G.); german.olivares@upc.edu (G.O.-P.); h.yang@yznu.edu.cn (H.Y.); qi.liu@henu.edu.cn (Q.L.); 2 IEEC, 08034 Barcelona, Spain; roma@ieec.cat; 3 GNSS Research Center, Wuhan University, Wuhan 430079, China; 4 Department of Physics and Mathematics, University of Alcalá, 28801 Alcalá de Henares, Spain; 5 Instituto de Ciencias del Espacio (ICE-CSIC, IEEC), Campus Universitat d’Autonoma de Barcelona, Carrer de Can Magrans, 08193 Cerdanyola del Vallès, Spain; 6 ESA, ESTEC, 2201 AZ Noordwijk, The Netherlands; raul.orus.perez@esa.int; 7 UPC-TALP, 08034 Barcelona, Spain; enric.monte@upc.edu; 8 School of Electronic Information and Engineering, Yangtze Normal University, Chongqing 408100, China; 9 College of Geography and Environmental Science, Henan University, Kaifeng 475004, China

**Keywords:** global electron content, space weather index, global navigation satellite systems

## Abstract

The time evolution of the total number of free electrons in the Earth’s ionosphere, i.e., the Global Electron Content (GEC), during more than two solar cycles is analyzed in this work. The GEC time series has been extracted from the Global Ionospheric Maps (GIMs) of Vertical Total Electron Content (VTEC) estimated by UPC-IonSAT with TOMION-v1 software from global GPS measurements since the end of 1996. A dual-layer voxel-based tomographic model solved with a forward Kalman scalar filter, from dual-frequency carrier GPS data only, provides the so-called UQRG GIM after VTEC kriging interpolation, with a resolution of 15 min in time, 5° in longitude and 2.5° in latitude. UQRG is one of the best behaving GIMs in the International GNSS Service (IGS).In this context, the potential application of the GEC spectrum evolution as a potential space weather index is discussed and demonstrated.

## 1. Introduction

The Global Electron Content (GEC) can be defined as the total number of free electrons in the ionosphere and plasmasphere up to the Global Positioning System (GPS) satellite altitude of 20,200 km [1,2]. The GEC can be computed by integrating the global ionospheric maps (GIMs) of VTEC on the overall ionosphere [3]. The GIMs are routinely computed from worldwide Global Navigation Satellite System (GNSS) multifrequency measurements by different analysis centers in the context of the International GNSS Service [4,5,6].

In order to be compared with the GEC we can consider the K-index [7], which quantifies disturbances in the horizontal component of Earth’s magnetic field with an integer in the range 0–9 with 1 being calm and 5 or more indicating a geomagnetic storm. The Kp-index is calculated by combining the data from multiple magnetic observatories around the world to determine the global planetary index.

Motivated by previous studies of specific geomagnetic storms where the GEC seems to evolve correlated with geomagnetic indices [8], GEC and Kp are compared in this work in both the temporal and frequency domains. The study is focused on the GEC and Kp spectrum evolution on the 10 days time intervals centered at the 34 geomagnetic events with Kp ≥ 7.5 from 2000 to 2020.

**Figure 1:**
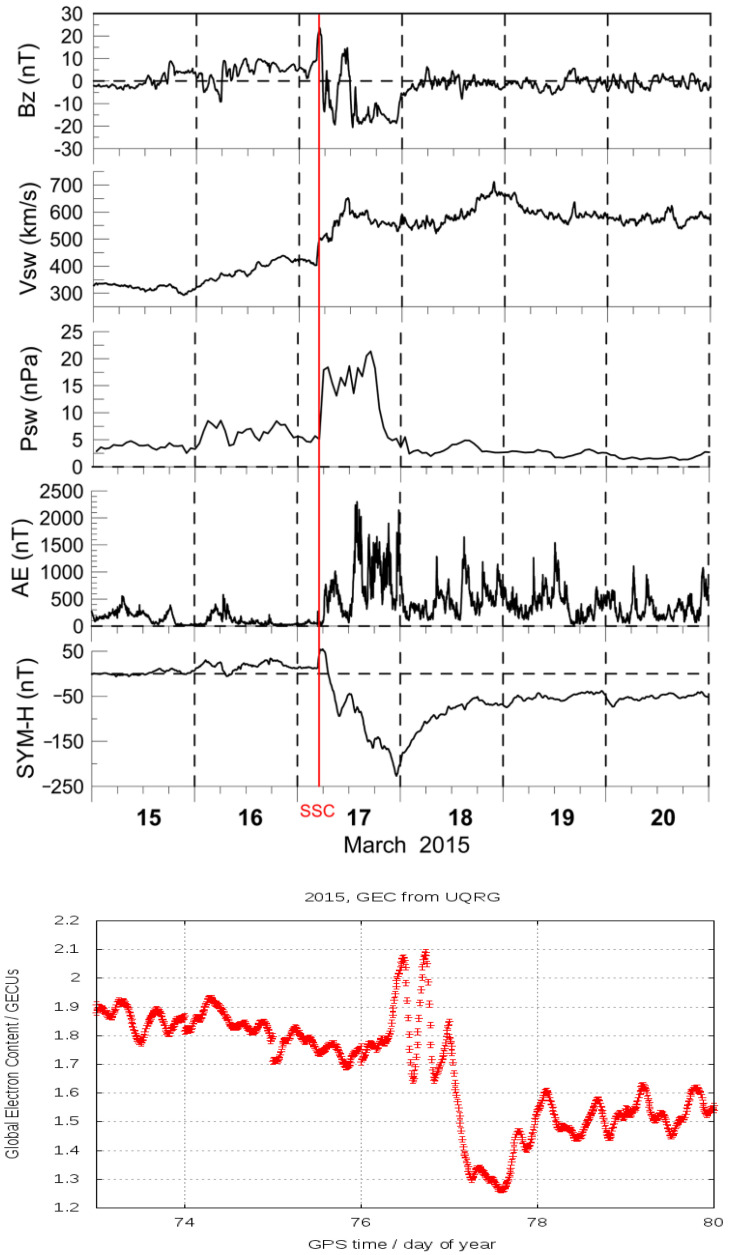
(**Top plots**): Space weather indices, $B_{z}$, $V_{sw}$, $P_{sw}$, AE and SYM−H vs. time, in days of March 2015, around the St. Patrick 2015 geomagnetic storm (see Sudden Storm Commencement indicated by the vertical red line). (**Bottom plot**): GEC vs. time in days of year 2015, and with the same time scale than top plots (reproduced from [8]).

Our first motivation for the present work was the apparent correlation found between some space weather indices and GEC time evolutions around the St. Patrick 2015 geomagnetic storm [8], as can be seen in Figure 1.

This apparent relationship of the GEC (hereinafter *G*) vs. SW indices has been studied in previous works under different approaches. Indeed, Ref. [9] summarized the GEC storm time modeling, from the JPL GIMs from 1999 to 2014, associated with variability of smoothed and normalized Auroral Electrojet index (positive correlation). The study considers the DGEC values taking the hourly ratio of instant GEC to 7 preceding days median.

Moreover, the authors of [10] studied 90 storm events from 1999 to 2011 from IGS GIMs in a different way. They conclude that the ratio $G/G_{qt}$ is closely correlated with geomagnetic Kp index (positively) and time weighted Dst index (negatively) and also affected by F10.7, where $G_{qt}$ is the 6 quiet days’ smoothed average GEC (Kp < 3.0).

More recently the authors of [11], with an approach similar to that used by [12,13], studied the geomagnetic storm of 7–9 September 2017 with the UPC-IonSAT UQRG GIMs: $\Delta G=G-G_{q}$ shows a clear correlation with SW indices like Kp and SYM-H (where the quiet time GEC, $G_{q}$ is obtained by using the three quiet days before the storm on which the Kp index is below 4).

In order to dispose of a most general view of GEC and Kp variability, not restricted to the given reference time windows for detrending (like the three ones of the mentioned recent works), we have performed a systematic comparison of the overall spectrum time evolution of GEC and Kp.

## 2. Methodology

The GEC time series for this study has been extracted from the GIMs of VTEC estimated by UPC-IonSAT with TOMION-v1 software from global GPS carrier phase measurements (i.e., not affected by pseudorange noise, multipath or differential code biases, see for instance [14]) since the end of 1996. An spectral analysis of the GEC time series is presented and, afterwards, the spectrograms have been computed and studied around major geomagnetic events.

### 2.1. How Is the GEC Computed

In order to compute the so-called UQRG GIM, first, a dual-layer voxel-based tomographic model is solved from dual-frequency carrier GPS data only by means of a forward Kalman filter; and, secondly, the kriging interpolation is applied. It has a resolution of 15 min in time, 5° in longitude and 2.5° in latitude, and is one of the best behaving GIMs in the International GNSS Service, IGS ([4,5]).

We have considered the GEC, computed as the surface integral of the GIM VTEC, from 2000 to 2020 (almost two solar cycles) but downdated to the Kp cadence of 3 h. This provided us a total of 34 periods of 10-days to study, centered at the geomagnetic events with Kp ≥ 7.5 in such a time interval, only taking one single event when they are consecutive (separated by less than 3 days). Then the GEC and Kp, but specially their spectra time evolution, by applying a continuous wavelet transformation, are compared.

### 2.2. How Are the Spectrograms Computed

Spectrograms are obtained through a continuous wavelet transformation, by using the corresponding package under the R statistical programming language. The basic wavelet function is the Morlet form:

(1) $$\Psi \left(t\right)=\pi^{-1/4}e^{-\frac{t^{2}}{2}+i6t}$$

To transform a time series $x_{t}$, indexed on time *t*, we compute the scalar product of $x_{t}$ and the shifted (by a time τ) and scaled version of Ψ(t), with scaling factor *s*. The output of the transformation is:

(2) $$W\left(\tau ,s\right)=\sum_{t}x_{t}\frac{1}{\sqrt{s}}\Psi^{*}\left(\frac{t-\tau }{s}\right)$$

And the final results and corresponding figures shown below correspond to the wavelet power spectrum $\frac{1}{s}\;{\left|W\left(\tau ,s\right)\right|}^{2}$.

Since the time series are finite samples, the projection over the wavelet function is less significant for τ close to the boundary when the periods are large. This is represented in the figures below by a lighter color region.

## 3. Results

After performing the GEC spectral analysis, the comparisons between Kp and GEC are presented in spectral and time domains, and in both ways: qualitatively, by visual inspection, and quantitatively, by means of the two corresponding Pearson correlation coefficients.

### 3.1. Spectrum of the GEC Time Evolution

**Figure 2:**
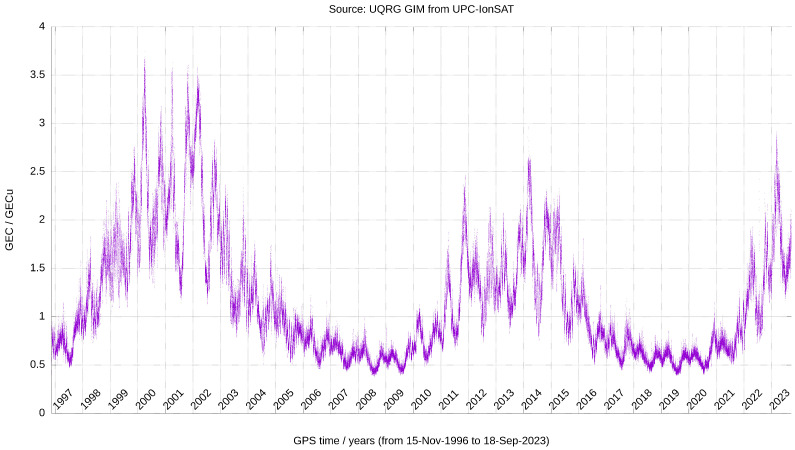
GEC, expressed in GECU, vs. time, in years, since the end of 1996 to September 2023. It has been computed from UQRG GIMs, with a time resolution of 15 min.

The spectral analysis of the GEC time evolution (Figure 2), computed every 15 min since the end of 1996 to September 2023, is summarized and compared with the previous results [15] in this section, following the decreasing order of spectral terms significance. We can see during more than two solar cycles, the annual and semiannual periods, with GEC maxima at the equinoxes, and minima at the solstices (which appear systematically low at the June one). And we can appreciate as well (in the resolution limit of the plot) that the higher frequency oscillations are associated with the solar rotation.

**Figure 3:**
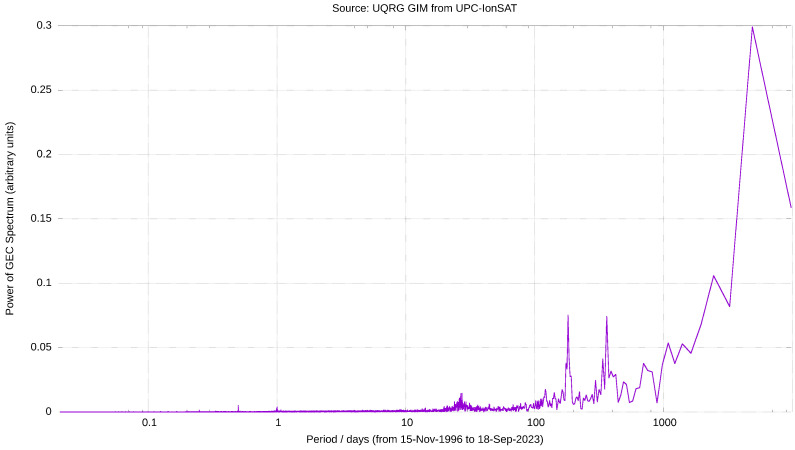
Absolute value of the GEC FFT, expressed in GECU, vs. logarithm of period in days, since the end of 1996 to September 2023. It has been computed from UQRG GIMs, with a time resolution of 15 min.

**Figure 4:**
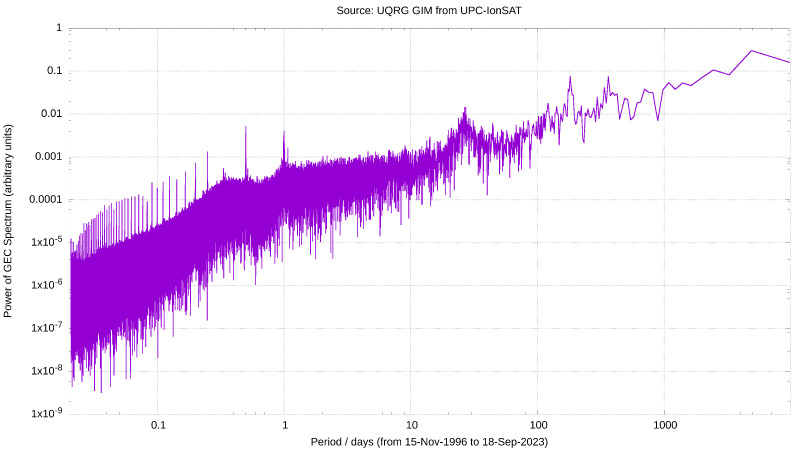
Logarithm of the absolute value of the GEC FFT, expressed in GECU, vs. logarithm of period in days, since the end of 1996 to September 2023. It has been computed from UQRG GIMs, with a time resolution of 15 min.

These four periods appear as the first, fourth, third and sixteenth most intense periods in the Fast Fourier transform (hereinafter FFT) of the GEC time series (Table 1), as it can be seen as well in the semilog and log-log representation of their module vs. period (Figure 3 and Figure 4, respectively), confirming the appearance under visual inspection. These GEC periods given by the FFT, after being applied to the almost one million GEC values derived from almost 27 years of 15 min GIMs, were also detected and reported in Figure 22 of [15], computed since June 1998 to end of 2007 from our UPCG GIMs at 2 h, i.e., a time series of around 40 thousands GIMs. In other words, in this study we analyze a set of GEC values more than 20 times larger than in [15] and with and extension almost three times larger (around 2.5 solar cycles vs. almost one), reaching to higher periods in the spectrum that were not available in the former study (see NA in Table 1), but confirming the most part of periods in the lower period interval.

Table 1 shows the (ordered) local maxima for the absolute value of the FFT of GEC, the periods where they occur and a estimation for the error on such periods. Since frequency is an integer index, the error on the period increases with the period value.Indeed, in Table 1 the complete set of the twenty most prominent periods in the temporal GEC spectrum can be found, and it shows that nine of them are in agreement with the above commented study (see the fifth column in the above-mentioned table). Moreover, it can be seen that other peak periods which are clearly defined in the GEC FFT: the one of 27.3±0.07 days is very well in agreement with the solar synodic rotation period (27.2753 days [16]), which affects the sunspot groups to the solar sunspots rotation period, the daily, half-daily and 6 h, the four of them are also observed in [15].

### 3.2. Qualitative Comparison of GEC and Kp

**Figure 5:**
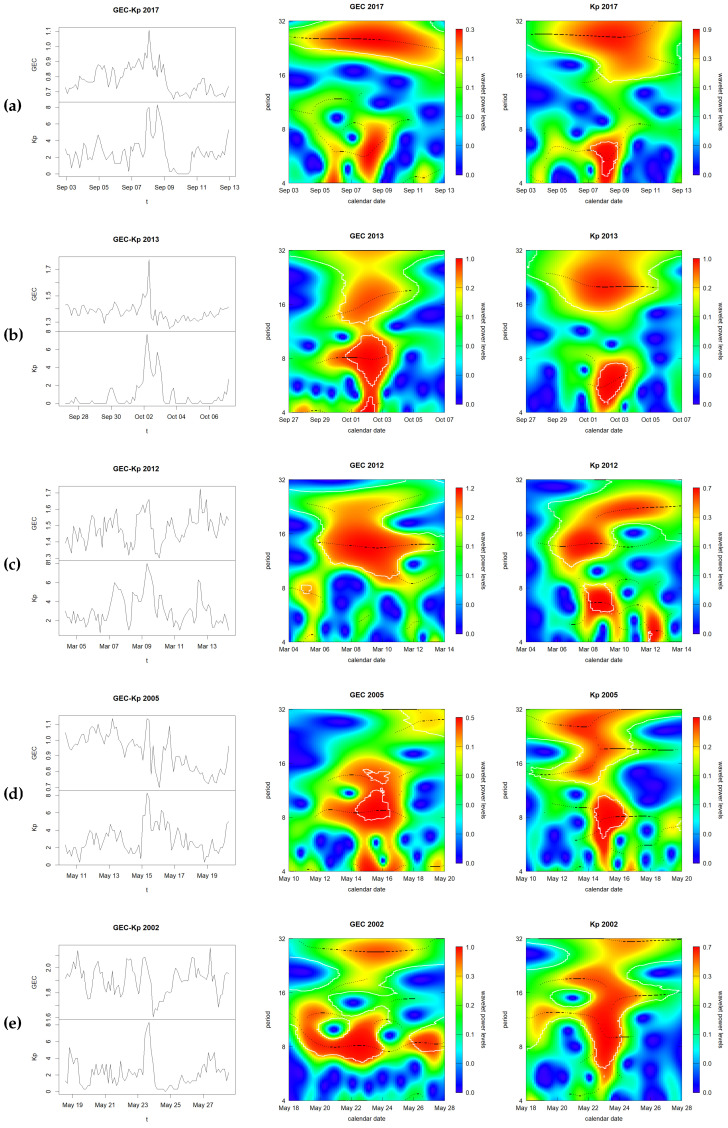
Representative examples of GEC and Kp time evolution (**top** and **bottom**, respectively of **left-hand plots**), GEC spectrograms (**central plots**) and Kp spectrograms (**right plots**), vs. time (horizontal axis, in days) and period (vertical axis, in multiples of three hours), with extremely high similarity (day 8 September 2017, **row a**), high similarity (day 2 October 2013, **row b**), mid similarity (day 9 March 2012, **row c**), low similarity (day 15 May 2005, **row d**) and extremely low similarity (24 May 2002, **row e**).

Examples of different qualitative levels of GEC and Kp spectra similarity can be seen in Figure 5, in terms of extremely high (day 2017-09-08, top plot), high (day 2013-10-02, second plot), mid (day 2012-03-09, third plot), low (day 2005-05-15, fourth plot) and extremely low similarity (2002-05-24, fifth plot).

**Figure 6:**
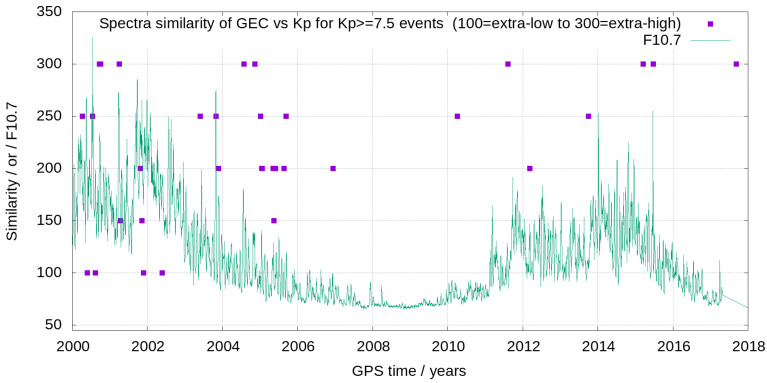
Evolution during more than one solar cycle of the level of qualitative similarity between GEC and Kp spectrograms (violet points), compared with the evolution of the F10.7 solar radio flux index (green line).

In summary, more than 50% of the GEC and Kp spectrograms during the geomagnetic events appear high or extremely high correlated by visual inspection, and only 21% of them appear low or extremely low correlated (see Table 2). In order to understand the context of low or extremely low spectral similarity between Kp and GEC, we represent the time evolution of Kp and GEC qualitative spectral similarity, besides F10.7 index, during the solar cycle in Figure 6. It can be seen that geomagnetic storms with Kp ≥7.5 presenting extremely low similarity between GEC and Kp spectra (four cases of 34) happen around the two extremely high F10.7 peaks (>200) of previous solar cycle.

This might be related with the correlation between the GEC and the F10.7 index, following [1,12]), which might affect the comparison with space weather indices, as it was pointed out by [11]. But before concluding about this potential explanations of this result, we have also performed, in the next subsection, a quantitative assessment of the degree of similarity between both, GEC and Kp distributions.

### 3.3. Quantitative Comparison of GEC and Kp

**Figure 7:**
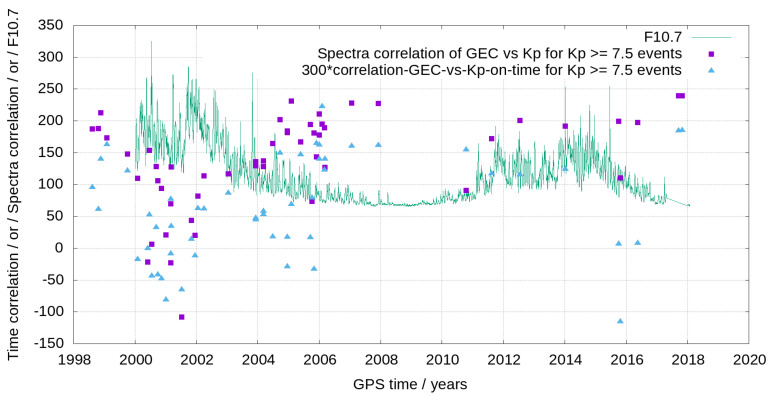
Evolution during more than one solar cycle of the correlation between GEC and Kp spectrograms (violet points) and the correlation between the GEC and Kp time series (light blue points). The evolution of the F10.7 solar radio flux index is also represented (green line).

It can be seen in Figure 7 that the quantitative similarity, correlation of both GEC and Kp spectra, confirms the qualitative one previously shown in Figure 6: indeed, a correlation decrease from predominant positive values up to +0.6 to values within the interval [−0.3,0.3], can be seen around the 2000–2002 solar cycle peak (coinciding with extremely low similarity in the previous plot). And higher correlation values, from 0.5 to almost 0.8, happen otherwise.

**Figure 8:**
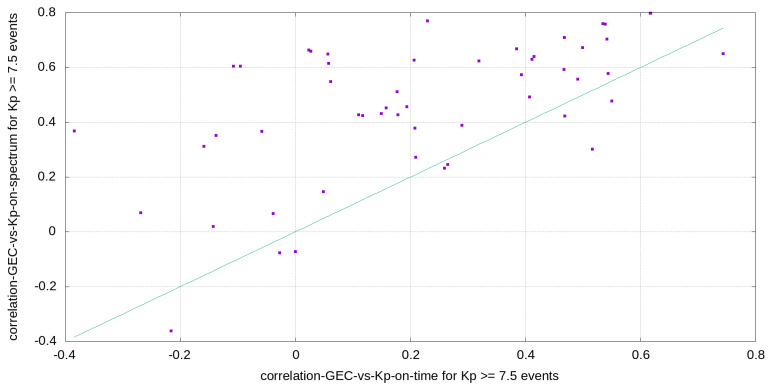
Points representing the correlation between GEC and Kp spectra vs. the corresponding correlation of GEC and Kp time evolutions during the geomagnetic events with Kp ≤ 7.5 (the straight line represent equal values for both).

It is interesting to see in the same Figure 7 the corresponding time evolution of the correlation of the GEC and Kp time series, which is systematically lower (as it is confirmed in the one-to-one spectrum vs. time GEC-Kp correlations represented in Figure 8), and in some periods beyond the 2000–2002 solar cycle peak, much lower. This result might be related with the time window used in the spectral analysis, less sensitive to latencies between GEC and Kp reactions to space weather conditions.

## 4. Conclusions

The GEC time series presents clear spectral signatures in periods which are confirmed, with regard to previous studies with a much reduced dataset, and extended in this research, based on +900,000 GIMs with +5000 VTEC pixel global values of each GIM (resolution of 15 min × 5° × 2.5° in time, longitude and latitude since the end of 1996 to September 2023). Motivated by previous works, the GEC and Kp geomagnetic index spectrograms have been systematically compared during time windows of 10 days around the 34 geomagnetic events, defined with Kp ≥7.5, from 2000 to 2020. After visual inspection, the majority of them (18 geomagnetic events) present a high or extremely high similarity, confirming the potentiality of using directly the GEC as SW index. However, there is a minority of GEC and Kp comparisons showing low or extremely low spectra similarity (7 of 34), which happened mostly around the strong solar flux epochs corresponding to the bimodal solar cycle peaks in 2000 and 2002. This result, quantitatively confirmed, is consistent with the potential contribution of high ionization epochs to the GEC spectra differently than the geomagnetic activity, pointed out by other authors in previous works.

In summary, we show in this work the higher correlation of GEC and Kp spectra, vs. the correlation of the GEC and Kp time series, during high geomagnetic activity periods. It can indicate the corresponding correlation of the extraordinary electron currents at high latitudes with the variability of electron content at large scale. Regardless of the potential common physical origin, the results strongly suggest the use of the GEC spectra to characterize space weather conditions.

## Figures and Tables

**Table 1:** The first twenty period values and error estimation in days, corresponding to the twenty highest absolute values of the GEC FFT modulus, are included in decreasing order. They have been computed from the complete GEC time series obtained from UQRG GIMs computed from UPC by the second author since the end of 1996 to September 2023. The best defined maxima from visual inspection of the corresponding plots, Figure 3 and Figure 4, are emphasized in bold. The periods also given in years and approximate solar rotation period (27.3 days) time units. The fifth column indicates whether the estimated period was already detected in [15] or not, from just one solar cycle of GIMs, including the periods too large to be detected in such a work (identified as Non Available, NA).

Period for 20 Highest GEC Local Maxima			|FFT(GEC)|	Is This Period Detected in [15]?
**/Days**	**/Years**	**/27.3 Days**		
4902±3000	13.42	179.56	0.299	NA
2451±600	6.710	89.78	0.106	NA
181.5±3.0	0.49692	6.64	0.075	**Yes** (180±6) days
363.1±13	0.9941	13.3	0.074	**Yes** (345±22) days
1089.3±122	2.9823	4.47	0.054	NA
1400.6±200	3.835	51.3	0.053	NA
338.1±11	0.9257	12.4	0.041	**Yes** (345±22) days
700.3±50	1.917	25.6	0.038	**No**
426.3±18	1.167	15.6	0.049	**No**
297.1±9	0.8134	10.9	0.024	**No**
490.2±24	1.342	18.0	0.023	**Yes** (551±56) days
121.0±1.5	0.3313	4.43	0.017	**Yes** (120±3) days
163.4±2.7	0.4474	5.99	0.017	**No**
222.8±5	0.6100	8.16	0.016	**No**
142.1±2	0.3890	5.21	0.015	**No**
27.3±0.07	0.07474	1.00	0.014	**Yes** (26.79±0.12) days
1.00298±0.0001	0.002746	0.03674	0.004025	**Yes** (1.00±0.01) days
0.49928±0.00003	0.001367	0.01829	0.001318	**Yes** (0.50±0.01) days
0.24929±0.00001	0.0006825	0.009132	0.001312	**Yes** (0.25±0.01) days
1.07266±0.0001	0.002938	0.03931	0.001610	**No**

**Table 2:** Summary of GEC and Kp spectrogram similarity determined by visual inspection.

GEC and Kp Spectograms Similarity	Number of Cases	Percentage of Cases
**Extremely high**	10	29%
**High**	8	24%
**Mid**	9	26%
**Low**	3	9%
**Extremely low**	4	12%

## Data Availability

The Global Ionospheric Maps datasets, baseline of this research, are openly available at the International GNSS Service main server https://cddis.nasa.gov/archive/gnss/products/ionex/ (only an initial free registration is required).
